# The axillary flap in oncoplastic resection of breast cancers located in the upper-outer quadrants: a new surgical technique

**DOI:** 10.1186/s12893-018-0467-3

**Published:** 2019-04-24

**Authors:** Daniele Bordoni, Pierfrancesco Cadenelli, Matteo Ornelli, Giuseppe Falco, Antonello Accurso, Antonio Gloria, Saverio Maietta, Nicola Rocco, Cesare Magalotti

**Affiliations:** 1 Department of Senology, Ospedale Santa Maria della Misericordia Urbino, Asur marche Area Vasta 1, Urbino, Italy; 2 Plastic and Hand Surgery Unit, ASST San Gerardo, Monza, Italy; 3 Department of Plastic Surgery, Marche Politechnic University, Ancona, Italy; 4 Breast Surgery Unit, IRCCS-Arcispedale Santa Maria Nuova, Viale Risorgimento 80, 42123 Reggio Emilia, Italy; 5 Department of Surgery, Breast Unit, University of Naples Federico II, Naples, Italy; 6 Institute of Polymers, Composites and Biomaterials, National Research Council of Italy, Viale J.F. Kennedy 54, Mostra d’Oltremare Pad. 20, 80125 Naples, Italy; 7 Department of Industrial Engineering, Fraunhofer JL IDEAS, University of Naples Federico II, P.le Tecchio 80, 80125 Naples, Italy; 8 Department of Clinical Medicine and Surgery, University of Naples “Federico II”, Via S.Pansini, 5, 80131 Naples, Italy

**Keywords:** Oncoplastic breast surgery, Breast Cancer, Surgical technique

## Abstract

**Background:**

The combination of breast conserving surgery (BCS) with plastic surgery techniques has provided a useful surgical tool matching the radicality of the oncological excision with the preservation of breast cosmesis. Even though BCS represents a good option for surgical treatment of tumors located in these quadrants, wide excisions often necessitate breast reshaping in order to avoid nipple areola complex (NAC) displacement and skin retraction. We present a new surgical technique to repair upper-outer quadrants’ defects following breast cancer excision using dermo-glandular flaps and an axillary adipo-fascial flap.

**Methods:**

During the period from January 2014 to December 2015, 168 patients with an upper-outer quadrant’s breast cancer have been treated in our Department. 83 women have been treated with the described oncoplastic technique and immediate contra-lateral symmetrisation and 85 women underwent standard BCS. We present surgical, oncological and cosmetic outcomes comparing our results with standard BCS.

**Results:**

At a mean follow-up of 27 months loco-regional recurrences in the two groups were comparable. Short-term complication rates were comparable between the two groups. Re-intervention rates for positive margins were significantly higher in the standard BCS group. The overall satisfaction with cosmetic outcome both assessed by the patient and the surgeon was significantly higher in the oncoplastic group.

**Conclusions:**

The proposed oncoplastic technique represents a safe and effective solution for reshaping that follows upper-outer breast cancer wide excision, achieving comparable complication rates, lower re-intervention rates for positive margins and better cosmetic results when compared with standard BCS.

## Background

The upper-outer quadrant of the breast is the most common location for breast cancer. Conventional Breast Conserving Surgery (BCS) is nowadays the standard surgical approach for the treatment of breast cancer located in the upper outer quadrants.

Although BCS followed by radiotherapy presents comparable survival rates with patients treated with mastectomy, it may hesitate in breast deformity in 20–30% of cases [1, 2]. Furthermore large series of patients treated by BCS show 20 to 40% of involved margins necessitating additional surgery to achieve oncological radicality [3, 4].

The possible distortion of breast shape and the following poor cosmetic result lead surgeons to develop different surgical techniques in order to overcome the aestethic discomfort for the patient [5]. The so-called oncoplastic breast surgery allows both a better cosmetic result and a wider tumor resection decreasing the risk of re-operation for positive margins [6–8]. Glandular resections regarding less then 20% of breast volume at the level of the upper-outer quadrants are usually well tolerated and do not hesitate in breast deformity. Even though BCS represents a good option for surgical treatment of tumors located in these quadrants, wide excisions often necessitate breast reshaping in order to avoid nipple areola complex (NAC) displacement and skin retraction. Effective methods such as the raquet mammoplasty, modifications of the Wise pattern for skin reduction and glandular displacement procedures have been proposed during the years with the aim of improving aesthetic outcomes after BCS [9–11].

We present a new surgical technique to repair upper-outer quadrants’ defects following breast cancer excision using dermo-glandular flaps and an axillary adipo-fascial flap. We present surgical and oncological outcomes comparing our results with standard BCS for the treatment of breast cancer located in the upper-outer quadrants.

## Methods

We retrospectively collected from our database all the women who underwent upper-outer quandrantectomies in our Department in the period from January 2014 to December 2015.

Women with a diagnosis of both invasive or in situ breast cancer, affected by both unifocal or multifocal lesions located at the level of the upper-outer quadrant have been enrolled in the study.

We divided our cohort of patients in two groups according with the surgical technique used to excise the breast cancer. The first group included 83 women who underwent our innovative oncoplastic technique and immediate contra-lateral breast symmeytrisation. The second group included 85 women who underwent a standard upper-outer BCS.

Women who previously underwent breast or axillary surgery or chest radiotherapy have been excluded. Patients with clinical pre-operative or histological intra-operative evidence of axillary lymph node involvement have been excluded as well, such as patients presenting skin involvement of the cancer, local recurrence, distant metastases or genetic mutations.

This study was approved by our institutional review board (Santa Maria della Misericordia Hospital Ethical Commitee # 2014/8769) and appropriate informed consent was obtained from all patients for the surgical procedures performed in the present study and for personal images use and publication.

Ultrasound examination, mammography, MRI and subsequent vacuum-assisted breast biopsy have been performed for all patients. All patients underwent a multidisciplinary approach, involving medical and surgical oncologists, plastic surgeons, breast radiologists, and radiation oncologists. In order to perform breast conserving surgery, we localized the lesions the day before surgery using Tc99m-MAA (Technetium 99 m- macro albumin aggregated); the sentinel lymph node was localized preoperatively by injecting Tc99m-nanocoll.

Positive margins were defined as presence of cancer cells at less than 1 mm from the specimen’s margin.

Post-operative radiotherapy (50 Gy on the breast) was administered in each case.

We assessed short-term post-operative complications (occurring less than 30 days after surgery), re-operations for positive margins of resection, local recurrences and cosmetic outcome (both patient-reported and surgeon-reported).

### Surgical technique

In the study group patients were marked the day before surgery with a vertical or inverted-T Wise pattern approach according to the balance between the expected tumor excision dimension and breast volume.

Antibiotic prophylactic therapy was administered to all patients according to hospital protocols.

The patient was placed in supine position with the arms abducted to 90 degrees and fixed on arm boards.

A two team approach was always planned in order to reduce operation room time and decrease the surgical stress for patients.

With a blade number 10 the incision was performed and all the skin within the preoperative markings was removed. Subsequently the skin over the tumor was undermined leaving a flap thickness of 3–4 mm. A wide tumor resection through the glandular tissue reaching the pectoralis major muscle fascia was performed.

Intra-operative frozen sections or radiographic examinations (when a cluster of microcalcifications was documented) of the specimen were performed in order to proceed to immediate re-excision if necessary.

Surgical clips were placed in the tumor bed following the tumor resection.

The sentinel lymph node biopsy was performed through the same skin incision.

Subsequent total skin undermining was performed in the medial and inferior quadrants of the breast to completely detach the gland from the skin.

An infero-lateral glandular flap was advanced upward, rotated and fixed with a 2/0 absorbable monofilament suture to an adipo-fascial flap mobilized from the axilla.

**Fig. 1:**
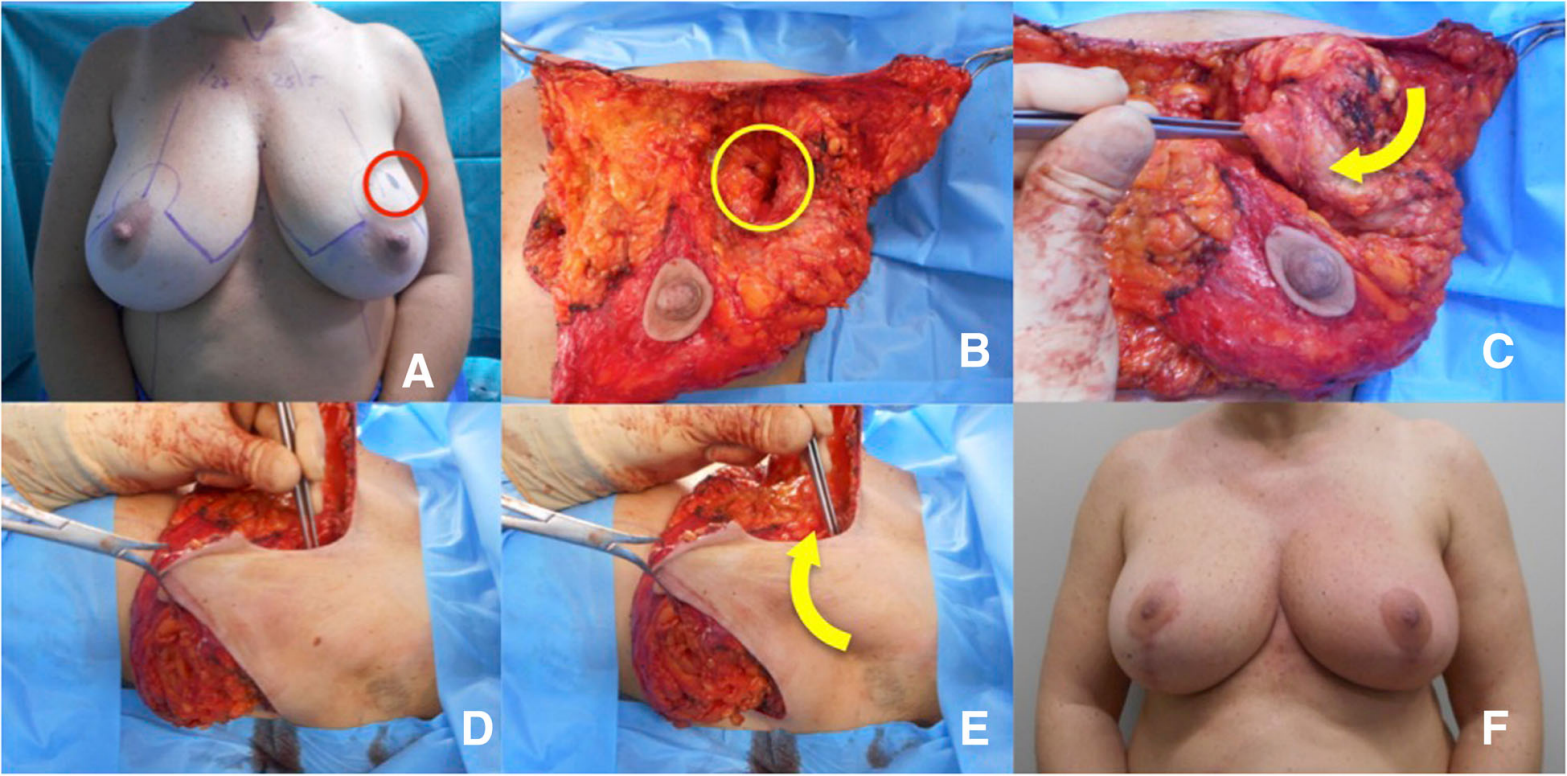
Case 1. **a =** Pre-operative drawings**; b =** Intra-operative view: the skin overlying the tumour is undermined in a mastectomy fashion (3–4 mm flap thickness) and a wide excision of the tumour down to the pectoralis fascia is performed (*yellow circle*)**; c, d, e =** Intra-operative view: the axillary flap is rotated to fill the defect in the Upper-Outer Quadrant (*yellow arrows*)**; f =** 1-year follow-up result

**Fig. 2:**
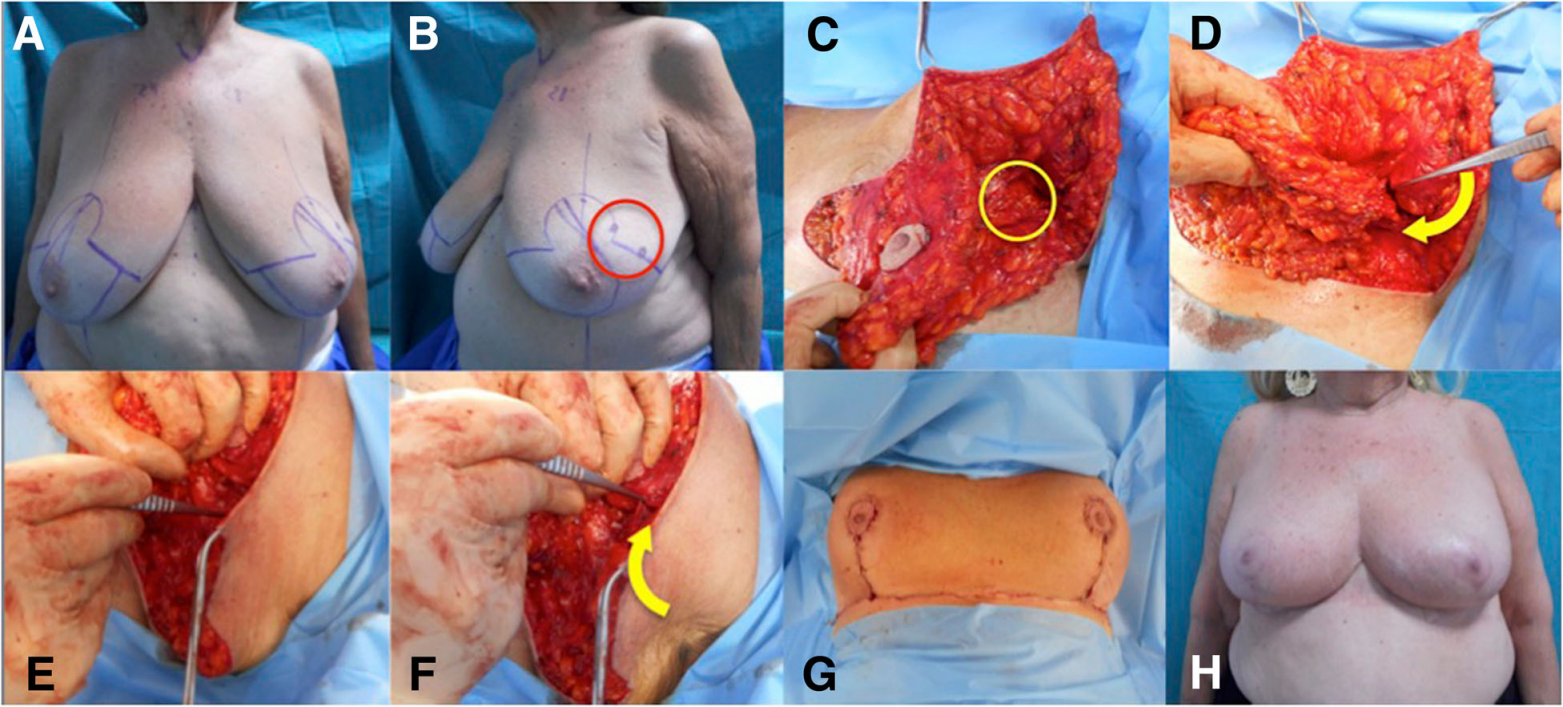
Case 2. **a, b =** Pre-operative drawings**; c =** Intra-operative view: the skin overlying the tumour is undermined in a mastectomy fashion (3–4 mm flap thickness) and a wide excision of the tumour down to the pectoralis fascia is performed (*yellow circle*)**; d, e, f =** Intra-operative view: the axillary flap is rotated to fill the defect in the Upper-Outer Quadrant (*yellow arrows*)**; g =** Immediate post-operative result**; h =** 1-year follow- up result

Then the residual glandular tissue of the upper portion of the breast could be approximated to the described flap and the nipple-areola complex was fixed in the desired position (Figs. 1 and 2).

When necessary, suction drains were positioned and then the suture was completed.

Contra-lateral symmetrization mammoplasty was always performed simultaneously.

Antibiotic therapy had been administered to all patients until drain’s removal.

In the control group patients underwent a standard upper-outer quadrantectomy. Sentinel Lymph node biopsy was performed through the same surgical incision.

## Statistical analysis

Variables were analysed using the chi-square test. A *p* value of 0.05 or less was considered statistically significant. All the analyses were performed using SPSS 22 software package (SPSS, Inc., Chicago, IL).

## Results

During the period from January 2014 to December 2015, 168 patients with an upper-outer quadrant’s breast cancer have been treated in our Department.

Eighty-three women have been treated with the described oncoplastic technique and immediate contra-lateral symmetrisation (study group) and 85 women underwent standard BCS (control group).

Patients’ Median age was 57.5 years (range 39–76) for the study group and 58.3 years (range 38–79) for the control group, median Body Mass Index (BMI) was 24.5 kg/cm2 (range 20.2–32.6) for the study group and 25.1 kg/cm2 (range 20.5–33.1) for the control group. The two groups were comparable for baseline characteristics and comorbidities. Patients’ comorbidities are presented in Table 1.

**Table 1:** Patients’ comorbidities

Comorbidities	OBS (83 patients) N of Patients (%)	sBCS (85 patients) N of Patients (%)	p*
Tobacco users	20 (24.1%)	13 (15.3%)	0.15
Diabetes	2 (2.4%)	5 (5.9%)	0.26
Obesity	2 (2.4%)	3 (3.5%)	0.67
Overweight	10 (12%)	12 (14.1%)	0.69
Hypertension	19 (22.9%)	23 (27.1%)	0.53
Dyslipidemia	8 (9.6%)	6 (7.1%)	0.56
Coronary Artery Disease	8 (9.6%)	8 (9.4%)	0.96

Surgical margins of resection were positive in 2 cases (2.4%) in the study group and in 9 cases (10.6%) in the control group (*p* = 0.03). All patients with positive surgical margins have been re-operated in both groups.

Mean time of follow-up was 27 months (range 16–39). During this period one patient (1.2%) has been re-operated for a local recurrence both in the study group and in the control group. Both patients presenting with a local recurrence were affected by Intermediate Grade (G2) Ductal Carcinoma In Situ (DCIS), completely excised at primary surgery with more than 5 mm free margin, and recurred as pT1N0 Invasive Ductal Carcinoma (IDC) at a follow-up of 25 months (in the study group) and 20 months (in the control group). Both patients underwent skin sparing mastectomy and breast reconstruction with tissue expander positioning.

All patients included in the study received post-operative radiotherapy. Adjuvant chemotherapy was administered to 23 patients (27.7%) in the study group and 26 patients (30.6%) in the control group (*p* = 0.79); hormonal therapy was provided to 57 patients (68.7%) in the study group and 60 patients (70.6%) in the control group (*p* = 0.68); Trastuzumab was provided to 13 patients (15.7%) in the study group and 14 patients (16.5%) in the control group. Oncological characteristics (T stage, Grading, ER, PgR, Ki-67 and Her-2) of both groups were comparable and are presented in Table 2.

**Table 2:** Oncological characteristics

Oncological Characteirstics	OBS (83 patients) N of Patients (%)	sBCS (85 patients) N of Patients (%)	p*
T			
Tis	9 (10.8%)	3 (3.5%)	0.07
T1a	11 (13.2%)	9 (10.6%)	0.60
T1b	16 (19.3%)	18 (21.2%)	0.76
T1c	38 (45.9%)	40 (47.1%)	0.88
T2	9 (10.8%)	15 (17.6%)	0.21
GRADE			
G1	18 (21.7%)	15 (17.6%)	0.50
G2	35 (42.2%)	32 (37.6%)	0.54
G3	30 (36.1%)	28 (32.9%)	0.66
ER positive	57 (68.7%)	52 (61.2%)	0.31
PgR positive	55 (66.3%)	54 (63.5%)	0.70
Ki-67 > 30%	44 (53%)	39 (45.9%)	0.36
Her-2 positive	13 (15.7%)	14 (16.5%)	0.89

We experienced a total of 18 (21.7%) short-term post-operative complications in the study group and 15 (17.6%) in the control group (*p* = 0.50). In the study group we experienced 4 wound dehiscences, 4 fat or glandular necrosis, 2 seroma formation, 2 hematoma, 4 marginal skin necrosis, 2 partial NAC necrosis.

In the control group we experienced 3 wound dehiscences, 3 fat or glandular necrosis, 3 seroma formation, 3 hematoma, 2 marginal skin necrosis, 1 partial NAC necrosis. Complication rates were comparable: wound dehiscence (*p* = 0.67), fat or glandular necrosis (p = 0.67), seroma formation (p = 0.67), hematoma (p = 0.67), marginal skin necrosis (*p* = 0.38), partial NAC necrosis (*p* = 0.56).

All complications in both groups have been managed conservatively with ultrasound-guided drainage of hematomas seromas and fat/glandular necrosis, use of hydrogel dressings for wound dehiscences and debridement and hydrogel dressings for minor skin and NAC necrosis.

The cosmetic outcome has been assessed by patients and surgeons following radiotherapy. The overall satisfaction with cosmetic outcome assessed by the patient was considered excellent in 45 cases (54.2%) in the study group, while in 32 cases (36.6%) in the control group (*p* = 0.02); the overall satisfaction with cosmetic outcome assessed by the surgeon was considered excellent in 48 cases (57.8%) in the study group and in 35 cases (41.2%) in the control group (*p* < 0.0001) (Table 3).

**Table 3:** Cosmetic outcome assessment

Cosmetic outcome	Self assessed				Assessed by surgeon			
	Excellent	Good	Fair	Poor	Excellent	Good	Fair	Poor
Oncoplastic Breast Surgery (*N*= 83)								
Breast symmetry	25 (30.1%)	48 (57.8%)	10 (12.1%)	0	35 (42.2%)	42 (50.6%)	6 (7.2%)	0
Nac symmetry	32 (38.6%)	49 (59%)	2 (2.4%)	0	40 (48.2%)	38 (45.8%)	5 (6%)	0
Breast shape	39 (47%)	42 (50.6%)	2 (2.4%)	0	40 (48.2%)	39 (47%)	4 (4.8%)	0
Scarring	25 (30.1%)	35 (42.2%)	21 (25.3%)	2 (2.4%)	28 (33.7%)	30 (36.1%)	23 (27.7%)	2 (2.4%)
Overall satisfaction	45 (54.2%)	30 (36.1%)	8 (9.6%)	0	48 (57.8%)	30 (36.1%)	5 (6%)	0
Standard Breast Conserving Surgery (*N*=85)								
Breast symmetry	10 (11.7%)	25 (29.4%)	41 (48.2%)	9 (10.6%)	10 (11.7%)	33 (38.8%)	33 (38.8%)	5 (5.9%)
Nac symmetry	9 (10.6%)	27 (31.8%)	34 (40%)	15 (17.6%)	12 (14.1%)	30 (35.3%)	40 (47.1%)	3 (3.5%)
Breast shape	29 (34.1%)	35 (41.2%)	15 (17.6%)	6 (7.1%)	31 (36.5%)	37 (43.5%)	9 (10.6%)	3 (3.5%)
Scarring	20 (23.5%)	31 (36.5%)	28 (32.9%)	6 (7.1%)	22 (25.9%)	37 (43.5%)	23 (27.1%)	2 (2.3%)
Overall satisfaction	32 (36.6%)	24 (28.2%)	23 (27.1%)	6 (7.1%)	35 (41.2%)	28 (32.9%)	17 (20%)	5 (5.9%)

## Conclusions

Breast Conserving Surgery (BCS) followed by radiotherapy has become the standard surgical approach for early stage breast cancer [12–15].

When adequate surgical margins are obtained local recurrence rates have been documented to range from 3.5 to 6.5% at 10-year follow-up [16, 17]. The introduction of Oncoplastic Breast Surgery (OPBS) allowed wider local excisions avoiding breast distortions that usually follows resections larger than 10–15% of total breast volume [18] .

Upper-outer quadrantectomies can be easily performed with standard BCS but breast deformity and NAC displacement may follow this procedure. Different oncoplastic approaches have been proposed during the years trying to overcome these complications. Small-sized breasts with early breast cancer can be effectively treated by Benelli mastoplasty: glandular rotation flaps combine good cosmetic results with satisfactory resections [19]. When wider resections are required emi-batwing excisions or racquet mammoplasties have been successfully proposed [20, 21]. These techniques consist in infero-medial flap reconstruction and supero-medial or inferior nipple repositioning with a skin resection of a crescent in combination with an ellipse. Although these techniques could achieve oncological radicality and good cosmetic results for the NAC position, the aesthetic result is affected by a long radial scar over the original tumor and a variable breast shape. Other authors propose the use of autologous tissue in order to fill the defect caused by upper-outer quadrantectomies. Even though flaps may provide pleasant aesthetic results, in our opinion these procedures should be reserved to selected cases because of their technical difficulty, their necessity of long operative time and issues related with donor site morbidity [22, 23]. Cutress proposed a modification of the Wise pattern reduction mammoplasty but using his technique the lower scar at the tumor side does not lie in the infra mammary fold but in the middle of the breast causing an important discomfort for the patient [24].

We aim to propose a reliable technique both respecting oncological radicality and preserving a pleasant breast contour. The large skin undermining and the breast reshaping performed using our technique do not determine NAC dislocations and irregular breast shapes.

Fitoussi and colleagues stated that the huge undermining could lead to increased rates of seroma, fat necrosis, bleeding and the NAC transposition may cause partial or total necrosis [25]. Other authors documented the relation between tumor location and the occurrence of complications. Tumors resection in the upper-outer quadrant tend to have higher complications rate when compared with other quadrants; the reason seems to be related with axillary lymphatics damage connected with tumor resection [26]. Our technique showed comparable short-term post-operative complication rates when compared with standard BCS and our rates are in line with those presented in literature.

Furthermore from an oncological point of view, we confirmed other data reported in literature evidencing that the oncoplastic approach reduces the number of re-operations for positive or close margins [27, 28].

We did not experience any delay for adjuvant therapies, either radio or chemotherapy, caused by a longer time of wound healing.

Some author suggest to postpone contra-lateral breast symmetrization after radiotherapy because of the risk of volume modification in the radiotreated breast [29]. We agree that fat necrosis or edema may derive from radiotherapy but we noted higher patients satisfaction levels when performing contra-lateral symmetrization in the same surgical time.

Our study presents the limitation of being retrospective and a longer follow-up could further confirm the oncological safety of the proposed surgical approach .

The proposed oncoplastic technique represents a safe and effective solution for reshaping that follows upper-outer breast cancer wide excision, achieving comparable complication rates, lower re-intervention rates for positive margins and better cosmetic results when compared with standard BCS.

## Abbreviations

BCS: Breast conserving surgery
BMI: Body mass index
DCIS: Ductal carcinoma in situ
ER: Estrogen receptors
IDC: Invasice ductal carcinoma
MRI: Magnetic resonance imaging
NAC: Nipple areola complex
OPBS: Oncoplastic breast surgery
PgR: Progesteron receptors
Tc99-MAA: Technetium 99 macro albumin aggregated
